# Malignant rhabdoid tumor of the omentum in an adult male: a case report and literature review

**DOI:** 10.3389/fonc.2023.1230021

**Published:** 2023-08-18

**Authors:** Xunjian Zhou, Zhi Duan, Ting Tao, Zhen Li, Ning Wang, Qimei Xu, Meiyan Wei, Zheng Zhong, Ran Liu, Qinghua Yin, Lixin Xiong, Hui Chen

**Affiliations:** ^1^ Department of Pathology, The Affiliated Changsha Hospital of Xiangya School of Medicine, Central South University, Changsha, Hunan, China; ^2^ Department of Infection and Immunity, The Affiliated Changsha Hospital of Xiangya School of Medicine, Central South University, Changsha, Hunan, China; ^3^ Department of Radiology, The Affiliated Changsha Hospital of Xiangya School of Medicine, Central South University, Changsha, Hunan, China; ^4^ Department of General Surgery, The Affiliated Changsha Hospital of Xiangya School of Medicine, Central South University, Changsha, Hunan, China

**Keywords:** malignant rhabdoid tumor, SMARCB1, omentum, pathological diagnosis, case report

## Abstract

Malignant rhabdoid tumors (MRTs) are rare tumors with high mortality rates and poor prognoses. MRTs occur mainly in the central nervous system, kidneys, and soft tissues, but rarely in the omentum. MRTs occur more commonly in infants and children and less frequently in adults. Here, we report the first observed case of MRT in an adult omentum. A 35-year-old man with abdominal distension and pain was admitted to the emergency department. Previously, several hospitals considered patients with cirrhosis who had not received active treatment. Computed tomography and magnetic resonance imaging revealed diffuse omental thickening and massive ascites. The surgery was performed at our hospital, and the pathological diagnosis was MRT with a SMARCB1(INI-1) deletion. Postoperatively, his symptoms improved, and he underwent five cycles of chemotherapy. However, 6 months after surgery, the tumor developed liver metastases, and the patient subsequently died. Primary MRT of the greater omentum is rare, and its pathological diagnosis usually requires extensive clinicopathological evaluation of various differential diagnoses and an appropriate work-up to exclude other malignancies associated with SMARCB1 deletion. At the same time, the lack of specific signs of omental MRT and its rapid progression should alert clinicians.

## 1 Introduction

Malignant rhabdoid tumors (MRTs) are rare and aggressive tumors that are more common in infants and young children than those in adults (1). In children, two-thirds of MRTs arise in the central nervous system, a disease known as atypical teratoid rhabdoid tumor (AT/RT). The rest occur in the kidneys, called MRTs, or in the soft tissues of other anatomical sites, sometimes called extrarenal or extracranial rhabdoid tumors (2). The absence of SMARCB1(INI-1) expression is an important feature of MRT. However, the pathological diagnosis of primary greater omentum MRT is difficult to distinguish from metastases of SMARCB1 deletion malignancies at other sites. Simultaneously, due to the lack of characteristic clinical manifestations, MRT can be easily missed by clinicians, thus delaying patient treatment. To date, no adult cases of omental MRT have been reported (3). Here, we report the first case of MRT in an adult omentum.

## 2 Case description

**Figure 1:**
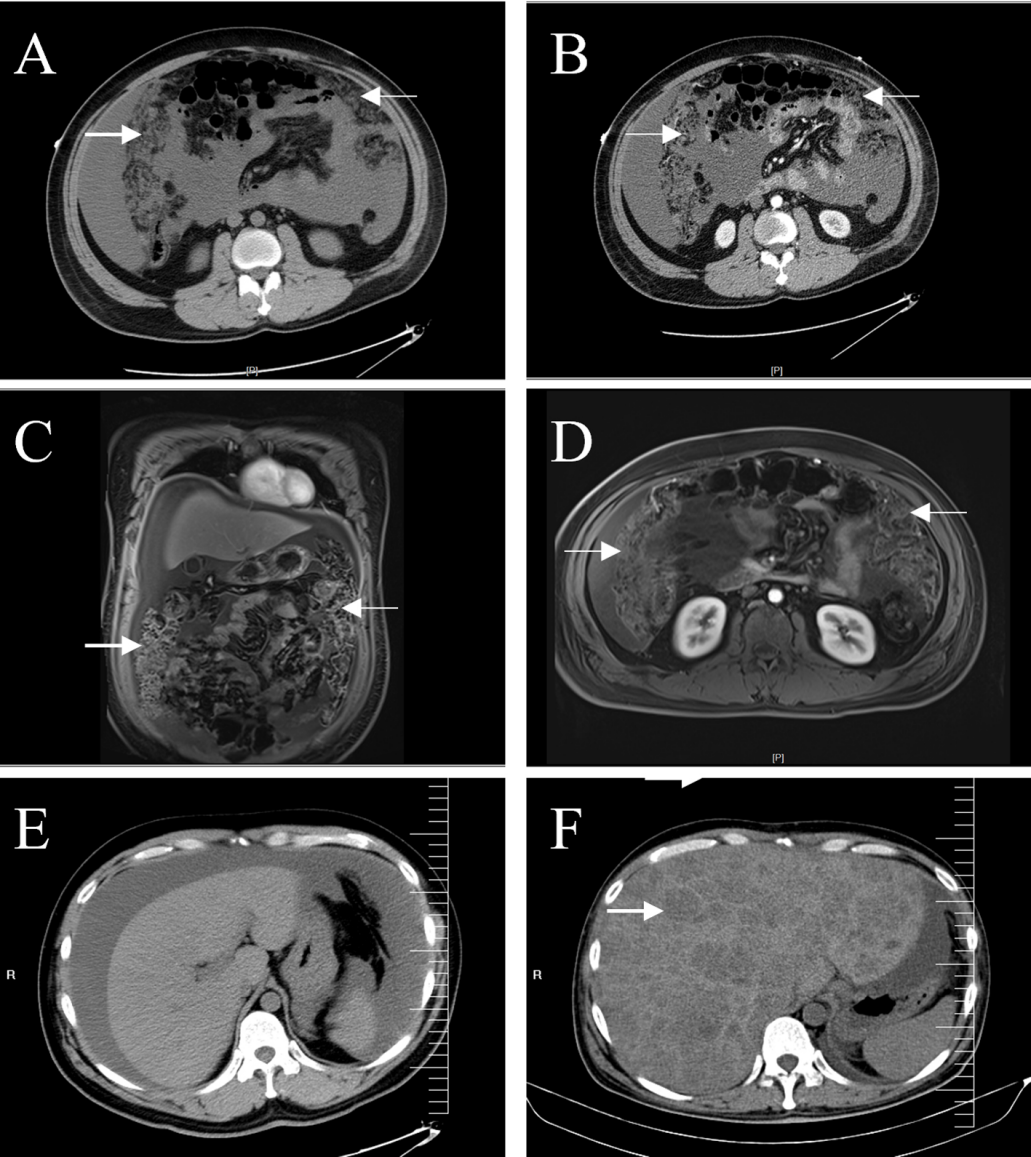
Abdominal CT and MRI. A plain **(A)** and enhanced **(B)** abdominal CT scans show a dense, blurred, and thickened omentum (arrows), with large amounts of fluid visible in the abdominal cavity. Coronal **(C)** and axial **(D)** enhanced T1-weighted images showed more clearly the irregularly thickened omentum (arrows). CT images of the liver showed no space-occupying lesions on admission **(E)**, and multiple low-density lesions (arrow) were observed in the liver six months later **(F)**.

The patient was a 35-year-old man who developed abdominal distension 1 month before presentation, and no significant abnormalities were found during a rectoscopy at a primary hospital. More than 10 d prior, he had visited two other hospitals for treatment because of bloating and abdominal pain. One hospital doctor considered the patient to have a fatty liver, while the other hospital doctors believed it to be cirrhosis; they did not further examine or treat the patient. Ten days prior, the patient visited our hospital with a gradual worsening of abdominal pain. Physical examination revealed mild tenderness in the lower left abdomen, no rebound tenderness, and positive abdominal mobility dullness. Abdominal computed tomography (CT) enhancement at our hospital showed severe ascites and diffuse nodular thickening of the greater omentum, the nature of which was not determined, and the possibility of cirrhosis was considered (**Figures 1A, B**). Abdominal magnetic resonance imaging (MRI) revealed irregular thickening of the peritoneum with massive ascites of unknown etiology (**Figures 1C–F**). No noticeable abnormalities were observed in the liver, gallbladder, bile duct, spleen, pancreas, kidney, bladder, prostate, or intestinal walls (**Supplementary Figure 1**). In addition, the father of the patient had a history of an ascending colon tumor, and the carbohydrate antigen 125 level was elevated to 200 U/mL (normal < 35 U/mL). A gastrointestinal endoscopy revealed polyps in the colon and several small duodenal ulcers. No malignant lesions were observed upon pathological examination. Viral hepatitis markers were negative, serum AFP and CEA were normal, and head and chest CT showed no abnormalities. Upon admission, the patient’s condition deteriorated rapidly, with incomplete intestinal obstruction, moderate iron deficiency anemia (hemoglobin, 81 g/L), and various severe electrolyte and metabolic disturbances. The patient’s basic condition was poor. After full communication with the patient’s family, the surgeon opted for emergency surgery. A large area of ascites was observed in the abdominal cavity during surgery. The entire omentum was enlarged like fish meat, which had partly invaded the bladder, abdominal wall, and mesentery.

**Figure 2:**
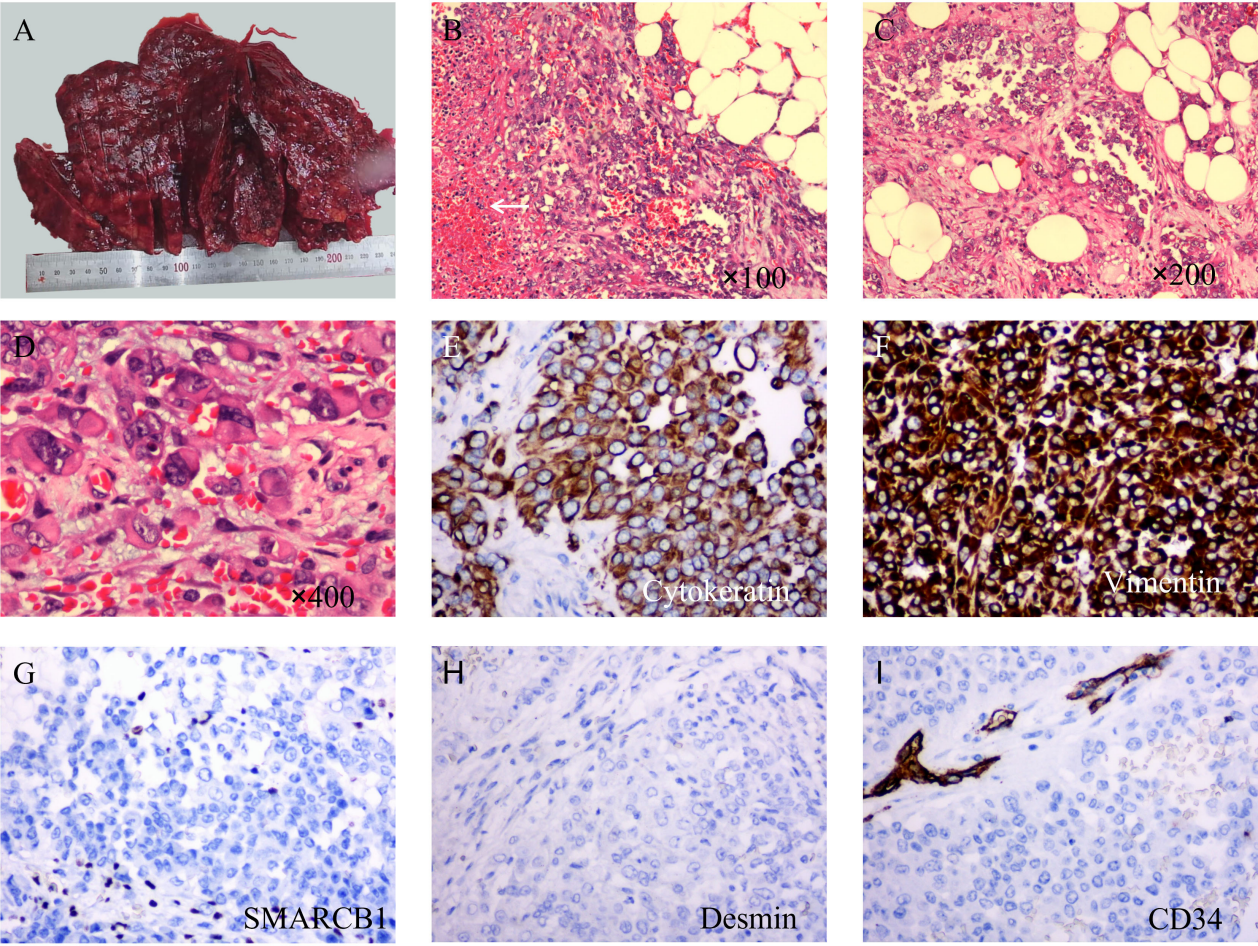
Macroscopic examination, hematoxylin & eosin staining, and immunohistochemical staining of tumors. **(A)** The surface of the tumor is gray-red and not very smooth. **(B)** At low magnification, the tumor cells showed a diffuse sheet-like growth pattern with poor intercellular adhesion, and necrosis (arrow). **(C)** In some areas, the tumor cells showed an adenoid growth pattern. **(D)** Under high magnification, the tumor cells are large in size, with abundant cytoplasm, an eosinophilic red color, large nuclei, deviated, vacuolated, and obvious nucleoli. **(E)** Immunohistochemical staining showed diffuse expression of cytokeratin (×200). **(F)** Vimentin is also diffusely positive and stains more deeply (×200). **(G)** SMARCB1(INI-1) was completely absent in the tumor cells while being positive in the surrounding cells. **(H)** Desmin is negative (×200). **(I)** CD34 was completely negative in tumor cells; however, peripheral vessels were positive (×200).

In general, the surface of the tumor was gray-red, and the cut surface was gray-red, gray-yellow, solid, and hard (**Figure 2A**). Microscopically, most of the tumor cells were distributed in solid sheets, and a small portion showed a glandular growth pattern. The tumor diffusely infiltrated the omental adipose tissue. The tumor cells were large in volume, rich in the cytoplasm, eosinophilic, and had large nuclei. The nucleus was located on one side of the cell, showing “rhabdoid” morphology, some of the nuclei were vacuolar, the nucleolus was obvious, and nuclear division was common (**Figures 2B–D**).

**Figure 3:**
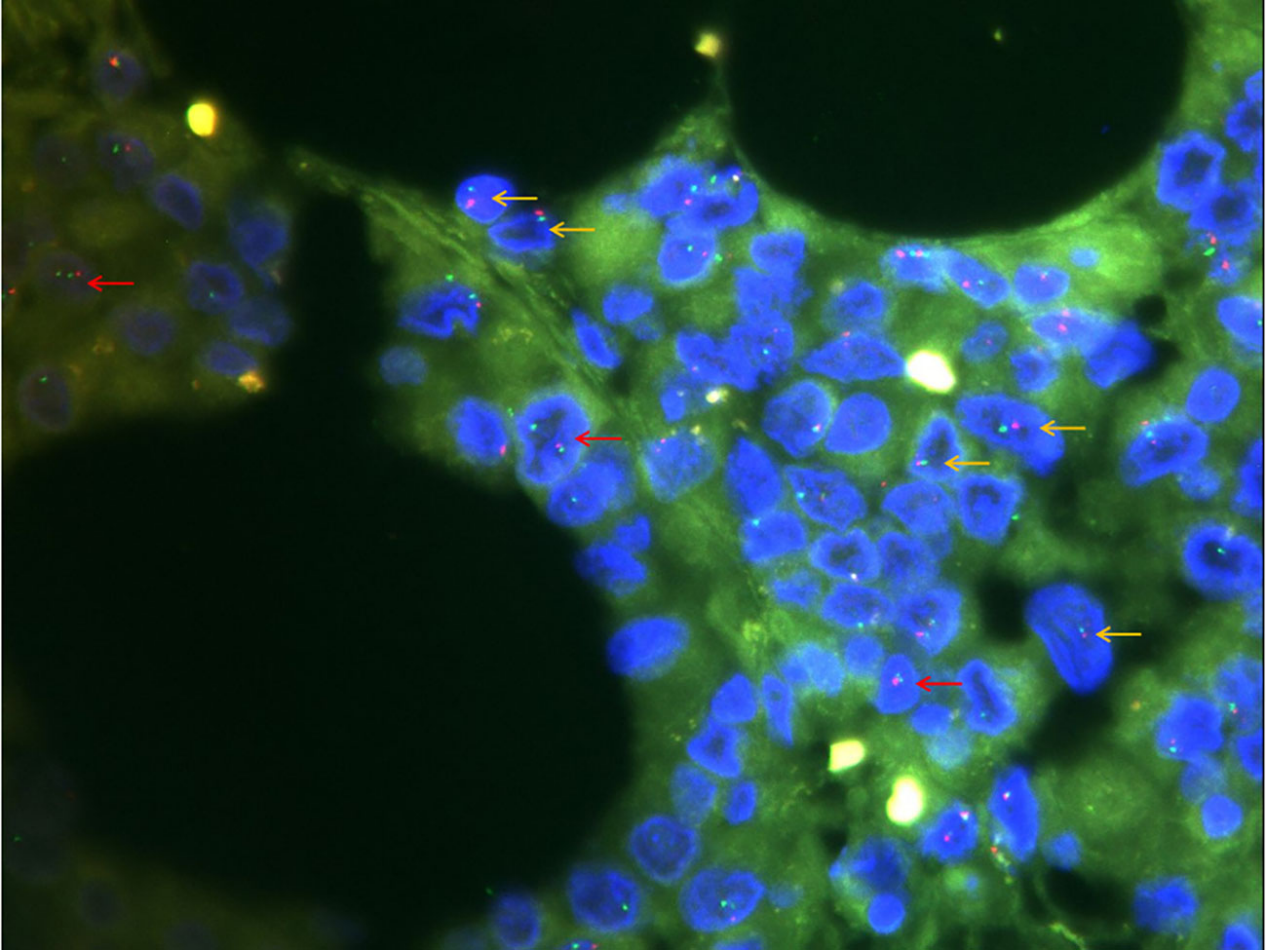
Fluorescence *in situ* hybridization detection of *SMARCB1* (×1000). Of the 100 cells counted, 20% had a red-green monomer (orange arrow) and 41% had 1R2G (red arrow), indicating *SMARCB1* heterozygous deletion.

Immunohistochemical and molecular detection: Immunohistochemical expression in the solid and glandular growth regions was similar, and the expression levels of cytokeratin, EMA, and vimentin were diffusely positive, but those of SMARCA4, desmin, and CD34 were negative (**Figures 2F–I**). Approximately 50% of the tumor cells were Ki67 positive. Importantly, SMARCB1 expression was lost. Similarly, a fluorescence *in situ* hybridization (FISH) assay revealed a *SMARCB1* deletion (**Figure 3**). In addition, immunohistochemistry of the tumor cells was positive for CK19, MUC5AC, MDM2, CDK4, BCL2, E-cadherin, and WT1. The tumors did not express CD31, ERG, D2-40, calretinin, actin, S100, SOX10, melana-A, HMB45, chromogranin A, synaptophysin, CD56, CD45, Sall-4, CD117, CD68, CK5/6, CK7, CK20, CDX2, SATB2, TTF-1, hepatocyte-1, glypican-3, Pax8, MUC2, or MUC6. Simultaneously, 507 fusion or rearrangement genes in common soft tissue tumors, including Ewing’s sarcoma and synovial sarcoma, were examined; however, no significant genes were found. Ultimately, the patient was diagnosed with MRT based on the results of auxiliary examinations.

**Figure 4:**
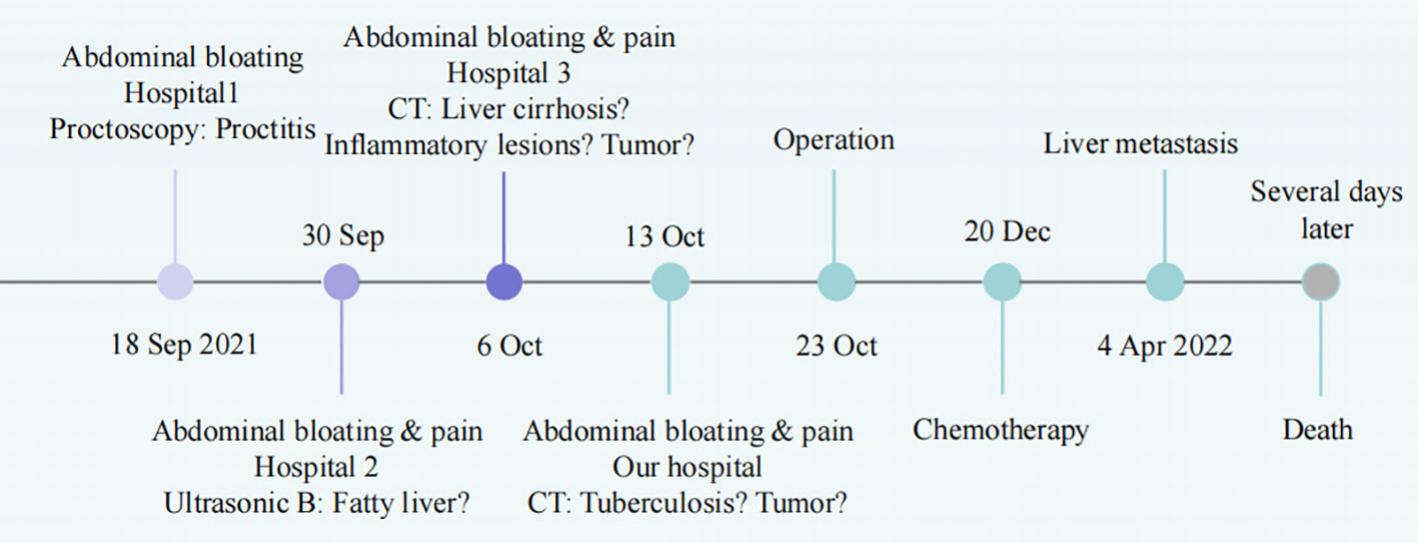
The timeline of diagnosis and treatment.

After two surgical debulking procedures, the patient was administered five cycles of ifosfamide + etoposide + mesna chemotherapy, and the ascites decreased. Unfortunately, 6 months after surgery, an MRI revealed metastases to the patient’s liver, and the patient died within a few days (**Figure 4**).

## 3 Discussion

### 3.1 *SMARCB1* and *SMARCB1*-deficient tumors

In 1998, MRT sequencing identified mutations, deletions, and other somatic alterations in *SMARCB1* (4). *SMARCB1*, located at 22q11.2, is one of the core subunit proteins of the ATP-dependent SWI/SNF chromatin remodeling complex (2, 5). SMARCB1, also known as INI1/BAF47, is ubiquitously expressed in the nucleus of normal cells and is an important tumor suppressor (4). Animal studies have shown that the disruption of *SMARCB1* expression leads to early embryonic death in mice (6). Although the immunohistochemical deletion of SMARCB1 is believed to contribute to the histological diagnosis of MRT, aberrant SMARCB1 expression has also been found in a variety of tumors, including epithelioid and synovial sarcomas (7). The abnormal protein expression of SMARCB1 can be divided into three modes: complete loss, mosaic expression, and reduced expression. Complete loss of SMARCB1 is observed in MRTs, epithelioid sarcomas, renal medullary carcinomas, epithelioid malignant peripheral nerve sheath tumors, myoepithelial tumors, extraosseous myxoid chondrosarcomas, pediatric chordomas, pancreatic undifferentiated rhabdoid carcinomas, sinus basaloid tumor carcinomas, and gastrointestinal rhabdoid carcinomas. Mosaic expression of SMARCB1 is observed in schwannomatosis, gastrointestinal stromal tumors, and fibromyxoma ossification. Decreased SMARCB1 expression is observed in synovial sarcoma (4).

### 3.2 Differential diagnosis

In this case, immunohistochemistry of the tumor cells showed SMARCB1 deletion, which should be differentiated from poorly differentiated carcinomas with SMARCB1 deletion. Ber-EP4 and MOC31 are believed to be present in a wide range of normal human and tumor epithelia and mature squamous cells and hepatocytes, playing a role in differentiated carcinomas and sarcomas (8). In this case, Ber-EP4 and MOC31 were only positive in small clusters of cells. More importantly, the clinician performed gastrointestinal endoscopy, chest and abdominal ultrasound, CT, MRI, and other examinations, but no space-occupying lesions were detected in the other organs; therefore, the above diagnosis can be excluded. Claudin-4 is a marker of epithelial differentiation that is expressed in almost all cancer types. Immunohistochemistry for claudin-4 was performed on 130 SWI/SNF complex-deficient tumors. Of these, 90% of MRTs (9/10) were claudin-4 negative, while 80% (16/20) of SWI/SNF complex-deficient undifferentiated carcinomas were claudin-4 membrane-positive (≥ 5% of cells), suggesting that claudin- 4 may play a role in differentiating MRT from *SMARCB1*-deficient differentiated carcinomas (9).

Second, the presented case must be differentiated from proximal epithelioid sarcoma. Proximal epithelioid sarcoma generally occurs in the body midline, is characterized by biphasic differentiation and *SMARCB1* gene deletion, and often has a morphology similar to that of MRT (10). Recent studies have shown that SALL4 and glypican-3 are expressed in some MRT cases and rarely in epithelioid sarcomas, but both were negative in the present case (10, 11). Compared with MRT, epithelioid sarcoma usually occurs in adults; therefore, epithelioid sarcoma is the most important differential diagnosis of the disease presented in this case. Morphologically, MRT usually presents as large, poorly adherent pleomorphic cells, whereas epithelioid sarcomas are often round or occasionally spindle-shaped with little pleomorphism (7). It is generally believed that at least 50% of epithelioid sarcomas express CD34. In addition, 38–68% (41/109, 19/28) of MRT cases were recently reported to be ERG-positive, while FLI1 and D2-40 are expressed in a higher proportion in epithelioid sarcoma (12–14). More importantly, Kohashi et al. analyzed 53 epithelioid angiosarcomas, of which 45 (84.9%) showed loss of SMARCB1 protein expression using immunohistochemical staining. Molecular testing of 39 of these patients revealed that only four (10.3%) had alterations at the DNA level sufficient to suppress gene expression, and the frequency of *SMARCB1* gene alterations was significantly lower than that in patients with MRT. In addition, MRT has a worse prognosis than epithelioid sarcoma (3, 15). Although the patient received active treatment, he still developed liver metastases and died after 6 months. Combined with the FISH detection of *SMARCB1* deletion and negative immunohistochemistry results for CD34 and ERG, the presented case is more in line with MRT.

Finally, the reported case must be differentiated from other tumors with biphasic differentiation, such as synovial sarcoma and malignant mesothelioma, which are features of MRTs. Synovial sarcomas originate from unknown pluripotent stem cells that differentiate bidirectionally into mesenchymal and epithelial cells. Synovial sarcomas tend to grow in the extremities, usually near large joints (7). Poorly differentiated synovial sarcomas can also exhibit a “rhabdoid” morphology, and reduced SMARCB1 expression can be observed in synovial sarcomas (16). Although the tumor cells of poorly differentiated synovial sarcomas are large, round, epithelioid, and frequently mitotic, they lack the significant pleomorphism of MRT tumor cells. In addition, immunohistochemical staining showed complete deletion of SMARCB1 expression rather than reduced expression, and no SYT-SSX fusion gene was detected, which is a relatively specific molecular change in synovial sarcoma; therefore, synovial sarcoma was excluded (17). Biphasic malignant mesothelioma usually has both epithelioid and sarcomatoid cells and usually does not have a SMARCB1 deletion. However, a rare case of malignant mesothelioma has recently been reported, with morphological features very similar to those of malignant rhabdomyoma and immunohistochemical staining showing a SMARCB1 deletion (18). Importantly, the rare malignant mesothelioma case expressed various mesothelial cell markers. However, all mesothelioma markers were negative except for the focal immunohistochemical expression of WT1 in the case presented at our hospital, allowing biphasic malignant mesotheliomas to be excluded.

### 3.3 SWI/SNF complex-deficient neoplasms

Mutations in genes encoding the subunits of the Switch/Sucrose non-fermentable (SWI/SNF) chromatin remodeling complex are common and occur in nearly 25% of tumors (2). Approximately 95% of MRTs have *SMARCB1* mutations, and another 5% have *SMARCA4* mutations (2, 5).

Likewise, an inactivating mutation of *SMARCA4* is an important molecular driver (19). *SMARCA4*-deficient tumors, similar to *SMARCB1*-deficient tumors, often exhibit a “rhabdoid” morphology, and both tumor types encode subunits of the SWI/SNF chromatin remodeling complex. Nearly 90% of small cell carcinomas of the ovary and hypercalcemic types have biallelic inactivating mutations in *SMARCA4* but rarely in *SMARCB1* (20). Thoracic *SMARCA4*-deficient undifferentiated tumors are newly identified rare tumor entities that present as large compressive and infiltrating mediastinal, pulmonary, or pleural masses and are often associated with smoking. They are undifferentiated tumors composed of epithelioid or rhabdoid tumor cells with a high mutational burden. Similarly, an inactivating mutation in *SMARCA4* is an important molecular driver (19).

In addition to *SMARCB1* and *SMARCA4*, at least seven genes encoding SWI/SNF complex subunits have been identified to be repeatedly mutated in various tumor types, including *ARID1A, ARID1B, ARID2, SMARCA2, PBRM1, SMARCC1*, and *SMARCC2* (21). Among these, *ARID1A* is the SWI/SNF composite gene with the highest mutation frequency and was found to be mutated in nearly 50% of ovarian clear cell carcinomas and endometrioid carcinomas. The next most frequent SWI/SNF composite genes with mutations are *SMARCA4* and *PBRM1*, with *PBRM1* mutations found in 41% of patients with clear-cell renal cell carcinoma (2). Studies have shown that *SMARCB1* plays a role in various signaling pathways, such as the p16-RB, canonical WNT, sonic hedgehog, and polycomb pathways (4). Molecular inhibitors of related pathways, such as EZH2, HDAC, CDK4/6, and mTOR, have entered clinical trials for MRT. In particular, EZH2 inhibitors, which directly target the negative consequences of *SMARCB1* loss prevalent in MRT, may improve MRT prognosis (22). Significant research progress has recently been made in immunosuppressive therapy for treating many solid tumors. Some studies have shown that the tumor cell proportion score of PD-L1 is higher in some MRT cases than that in other malignancies, indicating that these patients may benefit from immune checkpoint inhibitors (23). However, in the current case, we neither tested for PD-L1 nor attempted PD-L1-targeted therapy.

### 3.4 Strengths and limitations

We report a rare case of MRT in the omentum of an adult male, which is the first reported case of MRT in an adult omentum. The patient had relatively complete examination data, treatment interventions, and prognostic information. However, whole-exon sequencing, which may provide information for studying the pathogenesis and pathological diagnosis of MRT, was not performed in this case.

### 3.5 Experience and lessons

The case reported in this study can provide useful insights for clinicians and pathologists. MRT of the omentum is rare; owing to the lack of characteristic signs and symptoms, it is easily missed and misdiagnosed. For patients with unexplained ascites, clinicians should consider the possibility of malignant tumors and actively improve the relevant auxiliary examinations. Although the morphology of MRT overlaps with that of other SWI/SNF complex-deficient tumors, it is necessary to identify MRT because of its poor prognosis. This requires a pathologist to conduct an extensive clinicopathological assessment of the differential diagnosis and perform appropriate examinations. The prognosis of MRT is poor, and conventional surgical treatment and chemotherapy have little effect on the patient’s prognosis. Therefore, the pathogenesis and treatment of MRT require further study.

## 4 Conclusion

In summary, we report the first case of MRT in an adult omentum. MRTs of the omentum are rare, and as they lack specific signs and symptoms, they can be easily overlooked or missed. As such, we believe this finding deserves the attention of clinicians. At the same time, the morphology of MRT overlaps with that of other tumors, especially those with SWI/SNF complex deficiency. Pathologists must conduct extensive clinicopathological assessments of the differential diagnosis and appropriate examinations to make an accurate pathological diagnosis. Most MRTs are accompanied by *SMARCB1* deletions, which may provide new research directions for the treatment of this tumor.

## Data availability statement

The original contributions presented in the study are included in the article/**Supplementary Material**. Further inquiries can be directed to the corresponding author.

## Ethics statement

Written informed consent was obtained for the publication of this case report.

## Author contributions

XZ completed the data collection and the writing of the first draft. HC came up with the initial idea and completed the molecular testing. ZD, TT, and QX assisted in data interpretation. ZL and NW revised the manuscript. MW and RL assisted in completing part of the immunohistochemical experiments. ZZ provided the images. QY and LX provides surgical and patient information. All authors contributed to the article and approved the submitted version.
