# Supplementary material for: Malignant rhabdoid tumor of the omentum in an adult male: a case report and literature review
Source: Front Oncol. 2023 Aug 18;13:1230021. doi: 10.3389/fonc.2023.1230021 (PMC10473875; doi:10.3389/fonc.2023.1230021)
Supplement: Supplementary file 1 [file DataSheet_1.pdf]

Supplementary Material

Malignant Rhabdoid Tumor of the Omentum in an Adult Male: A Case Report and Literature Review

Xunjian Zhou[1], Zhi Duan[1], Ting Tao[1], Zhen Li[1], Ning Wang[2], Qimei Xu[1], Meiyan Wei[1], Zheng Zhong[3], Ran Liu[1], Qinghua Yin[4], Lixin Xiong[4] and Hui Chen[1]*.

[1]Department of Pathology, The Affiliated Changsha Hospital of Xiangya School of Medicine, Central South University, Changsha, Hunan, 410000, China.

[2]Department of Infection and Immunity, The Affiliated Changsha Hospital of Xiangya School of Medicine, Central South University, Changsha, Hunan, 410000, China.

[3]Department of Radiology, The Affiliated Changsha Hospital of Xiangya School of Medicine, Central South University, Changsha, Hunan, 410000, China.

[4]Department of General Surgery, The Affiliated Changsha Hospital of Xiangya School of Medicine, Central South University, Changsha, Hunan, 410000, China.

1. Supplementary Figures 1.

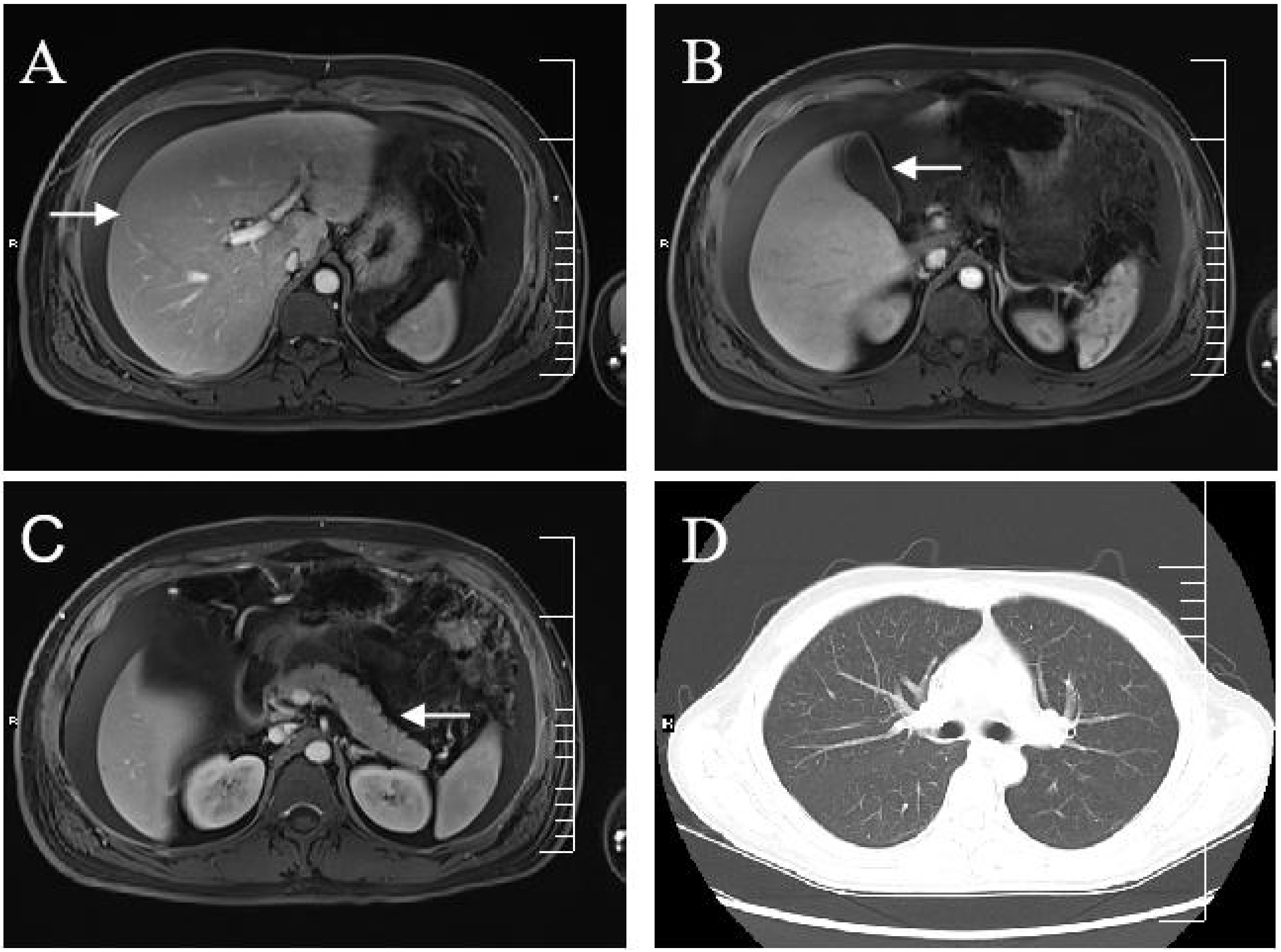

Supplementary Figure 1. Enhanced T1-weighted images of liver (A), gallbladder (B), and pancreas (C) showed no space-occupying lesions. No neoplastic lesions were found on chest CT (D).
